# Lipotoxicity as the Leading Cause of Non-Alcoholic Steatohepatitis

**DOI:** 10.3390/ijms23095146

**Published:** 2022-05-05

**Authors:** Marija Branković, Igor Jovanović, Marija Dukić, Tijana Radonjić, Svetlana Oprić, Slobodan Klašnja, Marija Zdravković

**Affiliations:** 1 University Hospital Medical Center Bežanijska kosa, Dr Žorža Matea bb, 11000 Belgrade, Serbia; igordusanov@yahoo.com (I.J.); marijadukic3@outlook.com (M.D.); tijana93@hotmail.com (T.R.); lana.opric@gmail.com (S.O.); slobodan.klasnja@gmail.com (S.K.); sekcija.kardioloska@gmail.com (M.Z.); 2 Faculty of Medicine, University of Belgrade, 11000 Belgrade, Serbia

**Keywords:** lipids, metabolism, NASH, NAFLD, liver

## Abstract

The emerging issues nowadays are non-alcoholic fatty liver disease (NAFLD) and its advanced stage non-alcoholic steatohepatitis (NASH), which further can be a predisposing factor for chronic liver complications, such as cirrhosis and/or development of hepatocellular carcinoma (HCC). Liver lipotoxicity can influence the accumulation of reactive oxygen species (ROS), so oxidative stress is also crucial for the progression of NASH. Moreover, NASH is in strong connection with metabolic disorders, and supporting evidence shows that insulin resistance (IR) is in a close relation to NAFLD, as it is involved in the progression to NASH and further progression to hepatic fibrosis. The major issue is that, at the moment, NASH treatment is based on lifestyle changes only due to the fact that no approved therapeutic options are available. The development of new therapeutic strategies should be conducted towards the potential NAFLD and NASH treatment by the modulation of IR but also by dietary antioxidants. As it seems, NASH is going to be the leading indication for liver transplantation as a consequence of increased disease prevalence and the lack of approved treatment; thus, an effective solution is needed as soon as possible.

## 1. Introduction

Non-alcoholic fatty liver disease (NAFLD) is an entity that includes different histological features from simple steatosis to non-alcoholic steatohepatitis with and without fibrosis [1]. It is characterized by steatotic changes in hepatocytes that are not a consequence of alcohol intake [2,3]. Histologically, simple steatosis and non-alcoholic steatohepatitis (NASH) are distinguished. In simple steatosis, more than 5% of hepatocytes show signs of steatosis but not signs of inflammatory damage and fibrosis. In contrast, in non-alcoholic steatohepatitis, more than 5% of hepatocytes are steatotic, but there are visible signs of ballooning hepatocytes: inflammation with the possibility of fibrosis [4,5]. Some studies have shown that the incidence of NAFLD in the world population is approximately 25%, but there are differences in terms of geographical area, ethnicity, race, and gender [3,6]. For example, the highest frequency was observed in the Middle East and the lowest in Africa [3,6]. In addition, in the United States, Latinos have the highest prevalence of the disease, while the lowest is present in Black population [6]. Regarding the difference between the genders, NAFLD and NASH are more common in men [1,6]. As mentioned earlier, NASH is an inflammatory form of NAFLD that occurs with progression in about 10–25% of patients with previously confirmed NAFLD [7]. About 20% of patients with NASH develop liver cirrhosis as a final consequence, which, in the most severe cases, requires transplantation [1,8]. It is believed that in the coming years, NASH, which according to previous studies is in third place, will overtake liver cirrhosis, which is caused by alcohol abuse and hepatotropic viruses and is the leading cause requiring liver transplantation in women [9]. In addition, patients with confirmed NASH have a significantly higher risk of developing hepatocellular carcinoma compared to the general population and even 12 times higher annual risk compared to patients with uncomplicated NAFLD [1,3,10].

The diagnosis of NASH is usually made incidentally given that patients are mostly asymptomatic [1]. The gold standard for diagnosis is liver biopsy, but due to the invasiveness of the procedure, non-invasive diagnostics are more often performed, which include abdominal ultrasonography and magnetic resonance imaging (MRI) of the abdomen but also special techniques, such as transient elastography or FibroScan [2,11,12,13]. There are also clinical scores and indexes (Fibrosis-4 index, NAFLD fibrosis score, aspartate aminotransferase to platelet ratio index (APRI)) that allow physicians to set diagnosis on time for treatment [14,15,16]. Based on previously known data, elderly patients and patients who have conditions that are included in metabolic syndrome (insulin resistance, type II diabetes mellitus, hypertriglyceridemia, visceral obesity) have a higher risk for progression of NAFLD to NASH [1,6]. Under these circumstances, lipids have a leading role in the development of NAFLD and NASH [17,18]. Over a long period of time, the pathogenesis of NAFLD was explained by a “two-hits hypothesis” [19]. The first hit in this theory is excessive fat accumulation in liver, which leads to the still-unknown second hit, which has the leading role in progression of NAFLD to NASH [19]. Latterly, this theory was substituted with the “multiple-hit hypothesis”, which is consistent with recent findings [19].

Like previously stated, insulin resistance is one of the predisposing factors for NAFLD pathogenesis. In this case, hyperinsulinemia promotes lipolysis and the release of free fatty acids and pro-inflammatory cytokines from adipose tissue [17,20,21,22,23]. In this way, an excessive amount of toxic lipids accumulates in the hepatocytes, which triggers a harmful mechanism known as “lipotoxicity” [24,25]. This mechanism leads to hepatocyte apoptosis, which can be prevented by mechanism of autophagy, which includes sending the excess of lipids to lysosomes for degradation [26,27]. However, in the case of hyperinsulinemia and excessive accumulation of lipids, autophagy may be disrupted, which potentiates hepatocyte apoptosis. This is one of the mechanisms by which NAFLD can progress to NASH [26].

On the other hand, releasing of pro-inflammatory cytokines from adipose tissue and consequential oxidative stress produce damage at the cellular level primarily to mitochondria and endoplasmic reticulum [25,28,29]. Due to all the above, a large number of studies dealt with the topic of lipotoxicity and the association of metabolic diseases with NASH [4,17,29]. In addition, cardiovascular diseases have been shown to be in a relationship with NAFLD and NASH and present the leading cause of death in patients with these conditions [11,30].

**Scheme 1:**
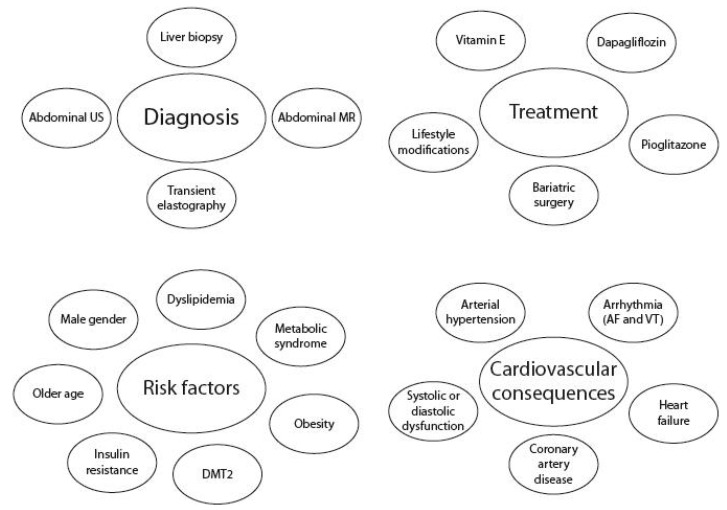
Diagnosis, treatment, risk factors, and cardiovascular consequences of non-alcoholic steatohepatitis.

There is currently no specific therapy for the treatment of NAFLD and NASH. The only approved therapeutic option represents lifestyle changes, including weight loss, dietary changes, and physical exercises [31,32,33] (Scheme 1). The aim of this paper is to present the importance of lipotoxicity in NAFLD and NASH and to accentuate the importance of new therapeutic approaches in NAFLD and NASH.

## 2. Pathophysiology of NAFLD and NASH

**Figure 1:**
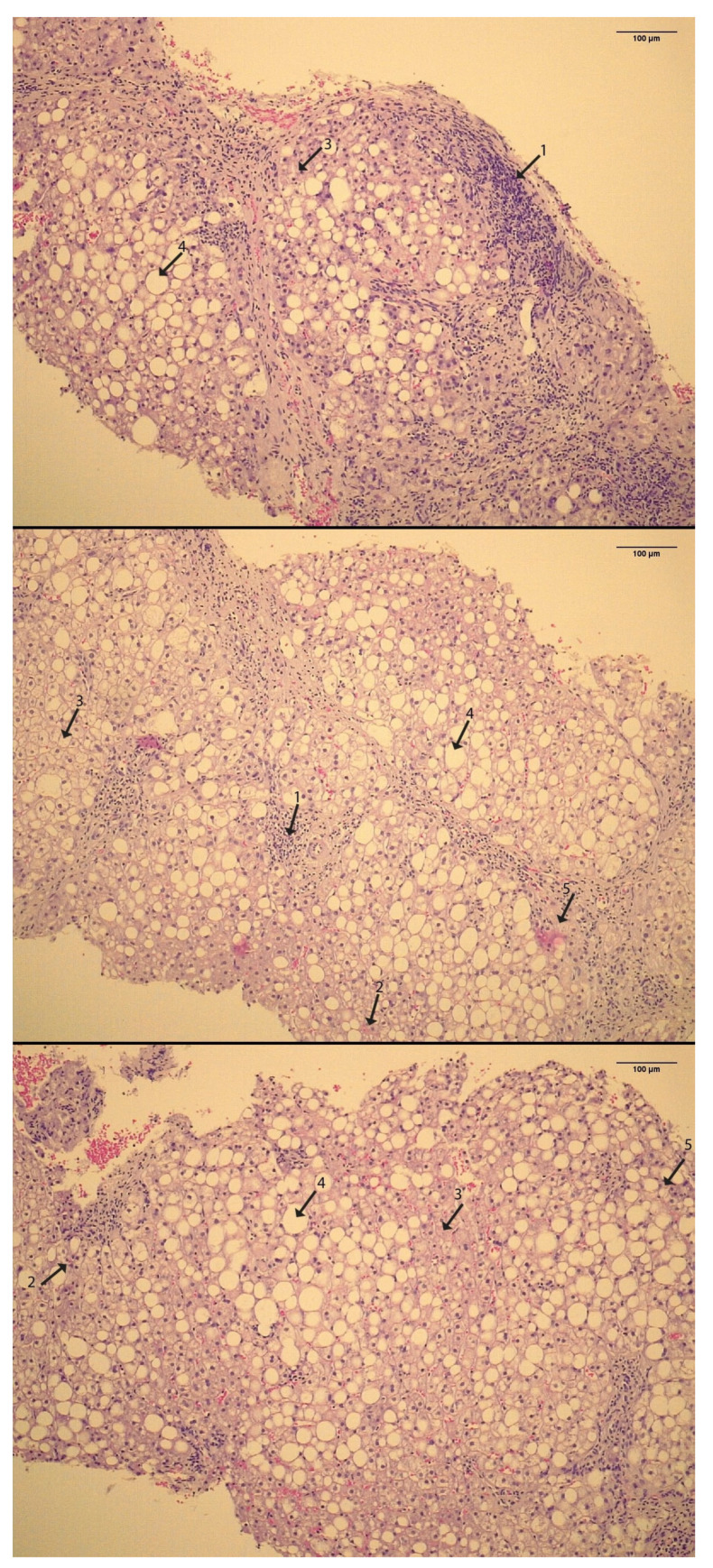
Liver biopsy of non-alcoholic steatohepatitis (NASH) stage 3 (severe fibrosis). Portal spaces are normal size or slightly dilated, connected by septa of connective tissue that sometimes surround parenchymal nodules. They are moderately to strongly infiltrated with lymphocytes and rare granulocytes. The parenchyma is partially nodularly transformed, and rare focal necrosis is present. Hepatocyte joists are focally thickened. Focal glycogenization of hepatocyte nuclei is present. Ballooned hepatocytes are numerous. Coarse-grained fatty change is diffuse. Kupffer cells multiply in the sinuses. There are numerous foci of perisinusoid necrosis. Hematoxylin and eosin (H&E) staining, 10 × Legend: 1, lymphocytes; 2, focal necrosis; 3, ballooned hepatocytes; 4, fat accumulation; 5, Kupffer cells.

Lipotoxicity is the one of the leading factors in the development of NAFLD and NASH. It is characterized by the accumulation of toxic lipids in cytoplasm of hepatocytes, which can lead to organelle dysfunction, cell damage, and eventually apoptosis [25] (Figure 1). It is associated with chronic inflammation seen in conditions that are included in metabolic syndrome respectively insulin resistance, type II diabetes mellitus, hypertriglyceridemia, and visceral obesity [34]. In addition to the onset, the role of lipototoxicity is important in the progression of NAFLD to NASH.

### 2.1. Harmful Lipids

Accumulation of harmful lipids in hepatocytes is closely related to the interaction between liver and adipose tissue. In the state of insulin resistance, lipolysis in adipose tissue is induced, i.e., the breakdown of triglycerides to free fatty acids. Free fatty acids through the systemic circulation reach the liver and accumulate there in the form of lipid droplets [35]. Triglycerides and cholesterol take the highest percentage in the construction of them [36,37]. This way is the most important for accumulating triglycerides in liver. Another mechanism by which the amount of triglycerides in the liver increases is their de novo synthesis [38]. This process is regulated by two transcriptional factors: sterol regulatory element-binding protein 1c (SREBP1c) and carbohydrate response element binding protein (ChREBP) [39]. Occurrence of de novo lipogenesis is promoted by glucose and insulin, which explains why patients with type II diabetes mellitus and insulin resistance often have NAFLD [40].

Some previous studies have shown that triglycerides have a less harmful and more protective role in case of accumulation in the liver. Yamaguchi et al. have shown that blockade of triglyceride synthesis by inhibition of diacylglycerol is associated with increased oxidative stress, inflammation, and fibrosis [41]. Similarly, it was proven in a study that was conducted by Alkhouri et al. [42]. Inhibition of adipose triglyceride lipase in mouse model reduces release of free fatty acids from lipid droplets [43]. As mentioned above, free fatty acids are created by triglyceride breakdown. According to number of carbon atoms, existence and place of different bonds and arrangement of hydrogen atoms free fatty acids are classified [42]. Several studies have indicated the importance of the harmful effect of unsaturated fatty acids, especially palmitate and stearate, in the development of steatosis and inflammation [44]. In contrast, monounsaturated fatty acids, such as oleate, are less harmful and even show a protective effect [45]. The protective role of monounsaturated free fatty acids is explained by reducing levels of pro-apoptotic proteins Bcl-2 interacting mediator of cell death (BIM) and p53 upregulated modulator of apoptosis (PUMA) [46]. The difference between impact of unsaturated and monounsaturated free fatty acids may be from different ability for promoting transformation of palmitate to triglycerides [47].

Unlike triglycerides, cholesterol can be harmful to the cell under conditions of increased liver load, for example, with increased fat intake [37]. In animal models, it has been proven that cholesterol can lead to the progression of simple steatosis in NASH [48]. The mechanism of this progression involves the activation of liver macrophages, resulting in chronic inflammation that characterizes NASH [49]. It is proven that especially free cholesterol is harmful, which was first concluded by Marí et al. [50]. Suggested mechanism of this toxic effect is change in fluidity of mitochondrial membranes and increased activity of tumor necrosis factor α (TNF-α) and Fas-dependent death signaling by accumulating in mitochondria [51]. Because of accumulation of free cholesterol in form of crystals in hepatocytes, after hepatocyte apoptosis, these crystals can get to Kupffer’s cells, where they can activate damage-associated molecular pattern (DAMP), which promotes activation of NLRP3 and other inflammatory patterns [52]. In addition, free cholesterol means the increasing vulnerability of hepatic stellate cells to transformative growth factor (TGF) β signaling, and in that way, it promotes fibrosis in NASH [53]. In the process of oxidation from free cholesterol, oxysterols are formed. These metabolites, as part of a for-now-unknown mechanism, can transform hepatic metabolism and precipitate pro-apoptotic, pro-inflammatory, and pro-fibrogenic pathways [54]. Some studies revealed that oxysterols can promote liver injury, causing mitochondrial damage, and because of the possibility to register them in serum of NAFLD patients, it can help to detect patients at high risk of progression of the disease [54].

In patients with NASH, high levels of ceramides are observed. Ceramides belong to family of sphingolipids, which participate in formation of cell membrane and can be made through de novo synthesis, a sphingomyelinase pathway, or from salvage pathway [55]. Furthermore, a diet rich in saturated fat can promote ceramide synthesis, which is confirmed in experimental NASH model. In fact, animals that were fed a diet rich in saturated fatty acids had higher levels of ceramides than those fed a diet rich in unsaturated fatty acids [56]. Under inflammatory conditions, especially under the influence of interleukin-1 (IL-1), interleukin-6 (IL-6), and TNF-α, ceramide levels increase, which means that they are associated with hepatocyte damage and ultimately their apoptosis [55].

In the pathogenesis of NAFLD and NASH, in addition to the mentioned harmful types of lipids, bile acids also participate. They are synthesized in hepatocytes from cholesterol in processes of hydroxylation and oxidation. This applies to primary bile acids, i.e., cholic acid and chenodeoxycholic acid. Further, there are also secondary bile acids, i.e., deoxycholic acid and lithocholic acid. Ursodeoxycholic acid is secondary bile acid that is present in small amount and today is used for treatment of cholestatic liver diseases [57]. For bile acids, it is proven that they disorganize cellular membranes by solubilization of fatty acids, cholesterol, and phospholipids [58]. Yet, little known to us about the role of bile acids in pathogenesis of NAFLD and NASH [59,60].

### 2.2. Organelle Damage—Endoplasmic Reticulum Stress and Mitochondrial Disfunction

Lipotoxicity promotes damage of cell organelles, most of all the endoplasmic reticulum and mitochondria [25]. The endoplasmic reticulum is the intracellular organelle responsible for protein synthesis, maturation, and transformation as well as lipid transport. Its function is regulated by special mechanism, which is called unfolded protein response [56]. This mechanism is susceptible to different metabolic changes, inflammation, and oxidative stress, which can lead to change of endoplasmic reticulum homeostasis. In this case, unfolded proteins production and accumulation activate unfolded protein response, whose role is to restore homeostasis [61,62]. If lipid accumulation continues, apoptotic pathways are activated, resulting in hepatocyte death. There are three main pathways in this case: ATF6 (activating transcription factor 6), PERK (RNA-dependent protein kinase-like ER eukaryotic initiation factor-2α kinase), and IRE1 (inositol-requiring enzyme-1), which are inactive under normal circumstances [61]. As mentioned, these pathways are apoptotic and endoplasmic stress can bring to hepatocyte death through c-Jun N-terminal kinase (JNK) signaling and C/EBP homologous protein (CHOP) activation [63,64]. The apoptotic pathways mediated by IRE1 phosphorylation include activation of JNK, which, in hepatocytes, triggers apoptosis and lessens oxidation of fatty acids, resulting in liver steatosis development [65,66]. In addition, this pathway exerts influence on pro-apoptotic molecules, such as Bax and Bak [67]. Additionally, activation of JNK and CHOP change calcium homeostasis, of which the endoplasmic reticulum especially sensitive because calcium is essential in protein folding, and its depletion causes apoptosis associated with endoplasmic reticulum stress [68]. How are toxic lipids associated with endoplasmic reticulum stress? When levels of diacylglyceride, phospholipids, free fatty acids, and free cholesterol are higher, it causes activation of endoplasmic stress through IRE1 and PERK pathways and increases expression of genes for lipid synthesis [69]. As stated earlier, calcium concentration has key role in endoplasmic reticulum homeostasis. Saturated free fatty acids, especially stearate and palmitate, can disrupt calcium homeostasis and cause activation of mitochondrial apoptotic pathways, which characterizes pathophysiology of NASH [44,70,71,72,73]. Endoplasmic reticulum stress is also described as one of the reasons for progression of NAFLD to NASH because of oxidative stress, which is the result [73].

Mitochondrial disfunction is recognized as the sign of lipotoxicity in patients with NAFLD and NASH ad even as a possible indicator of progression of NAFLD to NASH [24]. In a state of excessive accumulation of free fatty acids, which exists in NAFLD, some of mitochondrial pathways are over expressive, in particular in NASH patients: β-oxidation, respiratory chain, and hepatic tricarboxylic acid cycle (TCA) [74,75,76]. In conditions where there is a constant influx of free fatty acids, the β-oxidation process is intensified, due to which larger amounts of acetyl-CoA are formed, which can disrupt TCA in liver, deteriorate adenosine triphosphate (ATP) synthesis, and increase the formation of reactive oxygen species [74]. The most common way of causing mitochondrial damage is oxidative stress, most often mediated by peroxynitrate or peroxynitrite-derivative radicals [17]. The resulting mitochondrial dysfunction promotes the production of reactive oxygen species, cytokine release, and cell death [28,29]. Higher levels of oxidative stress have been shown to be associated with the progression of NAFLD to NASH. In addition, β-oxidation of larger amounts of free fatty acids may result in the occurrence of toxic lipids, such as ceramides, which can contribute to NASH pathogenesis [77].

Overall, the mechanism of lipotoxicity includes damage to cell organelles, most notably to endoplasmic reticulum and mitochondria. This occurs when the accumulation of lipids in hepatocytes exceeds the capacity of homeostatic mechanisms, and apoptosis signaling pathways (ATF6, PERK, and IRE1) are initiated; eventually, they can cause liver cell death through JNK signaling and CHOP activation.

### 2.3. The Role of Gut Microbiota in Lipotoxicity

The gut microbiota is made up of a large number of different microorganisms, but a significant number of them are bacteria, even more than 1000 species [78]. Their genes represent the microbiome, which is several hundred times larger than the human genome [78]. Gut microbiota is the main component of gut-liver axis that is realized through enterohepatic circulation [79,80]. Modifications in gut microbiota, also known as dysbiosis, which occur in the development of NAFLD can affect lipid metabolism, stimulate their accumulation in the liver and lipotoxicity. This is the result of oxidative stress that occurs as a consequence of the action of bacterial products but also the metabolism of free fatty acids in the liver, and both processes are mediated by inflammasome [81]. The gut microbiota is an important parameter of homeostasis of a healthy organism, and it is important for the body’s defense, i.e., the development of the immune system, but also the regulation of energy metabolism [82]. Therefore, all changes in the gut microbiota have an impact on the homeostasis of a healthy organism; i.e., they can have a role in the development of various diseases, including NAFLD [83]. The mechanisms are different. For example, most of the metabolic changes caused by dysbiosis are mediated by changes in metabolism of short-chain fatty acids. They can be detected in feces of NAFLD and obese patients [84,85]. Their importance is in steatosis development through different metabolic mechanisms, such as de novo lipogenesis, cholesterol synthesis, and changes of glucose metabolism [86]. Intestinal microbiota products can induce enhanced lipoprotein lipase (LPL) activity by inhibiting fasting-induced adipocyte factor (FIAF) that inhibits the activity of this enzyme. The result of this action is the accumulation of free fatty acids in the liver that are released from very-low-density lipoprotein (VLDL) [25]. Besides previously explained metabolic changes, dysbiosis could participate in NAFLD development and its progression to NASH by changes in intestinal permeability. In this way, the passage of bacteria and their metabolic products, for example, lipopolysaccharide, is facilitated, and thus, they enter the portal circulation more efficiently and manifest their effects [25,87,88]. Translocation of bacteria from intestinal lumen into the portal circulation increases the expression of Toll-like receptors on the surface of hepatocytes that enable the action of pro-inflammatory cytokines, especially TNF-α, interleukin-1β, and interferons [89]. The same effect is verified by free fatty acids, which are formed as products of bacterial metabolism, they activate the Toll-like receptor 4 [64]. This is important because it has been shown that reduced expression of Toll-like receptor 4 can reduce liver damage in the case of steatosis but also steatohepatitis [90,91]. The earlier-mentioned lipopolysaccharide, which is the major compound of membrane of Gram-negative bacteria, promotes low-grade inflammation after entering the portal circulation. It is shown in patients with NASH that levels of lipopolysaccharide are greater than in the patients with simple steatosis [92,93,94,95]. Lipopolysaccharide is recognized by Toll-like receptors, especially Toll-like receptor 4, thus initiating a cascade of synthesis of proinflammatory cytokines, mostly TNF-α, and promoting the onset of inflammation and oxidative stress [93,94,95]. Moreover, hepatic stellate cells are activated in this process, which means that liver fibrosis is promoted [96]. It has been proven that this is related to the progression of NASH.

## 3. Fibrosis—The Result of Continuous Inflammation

Chronic liver diseases of various etiopathogenesis most often lead to chronic inflammation resulting in liver damage. The most common consequence of chronic inflammation in this case is the formation of fibrosis [97]. Fibrogenesis occurs as a consequence of damage to liver parenchymal cells, and the process itself is controlled by endothelial cells and stellate and Kupffer cells, with the help of macrophages, dendritic, and mast cells. In the complex interaction of these cells, various pro-inflammatory and pro-fibrogenic mediators are synthesized in the damaged liver tissue [98]. In addition, the cells that produce the matrix, hepatic stellate cells that transform in myofibroblasts, are activated and participate in the development of fibrosis. Drivers for that transformation and activation of hepatic stellate cells are different. For example, the result of lipotoxicity in hepatocytes, among other things, is production and release of reactive oxygen species, which are recognized as stimuli for activation of stellate cells. Reactive oxygen species ensure paracrine activation of hepatic stellate cells. For example, interleukin-17 (IL-17) induces type I collagen production in stellate cells via the signal transducer and activator of transcription 3 (STAT3) signaling pathway [99]. Moreover, characteristic of NAFLD and NASH is a higher amount of free cholesterol, which is also a stimulus for fibrogenic actions of stellate cells [100]. In this way, the architecture of the liver tissue is changed by cumulation of a larger amount of connective tissue, which now replaces the functional liver tissue and leads to the loss of liver function [101]. The end effect of these reactions is cirrhosis, which is characterized by progressive deterioration of liver function followed by numerous complications [101]. Precisely because cirrhosis is a step towards liver transplantation, the importance of the mechanisms of fibrosis as well as its inhibition and regression has been recognized by experts. In about 37–84% of patients with NASH, fibrosis is verified, and it is related to poorer prognosis in these patients [102,103,104].

## 4. How to Diagnose NAFLD/NASH?

The diagnosis of NAFLD and NASH is commonly made accidentally because most patients are asymptomatic at the moment of diagnosis [1]. Several different methods, both laboratory and radiological, are used to assess the existence of NAFLD and NASH, but none of them is reliable enough or could replace a liver biopsy in a definitive diagnosis [105]. Transaminases as one of the indicators of liver function are not reliable in making a diagnosis because up to 80% of patients may have normal levels at diagnosis [106]. In patients with progressive NAFLD, alanine aminotransferase (ALT) is more often elevated, but normal values of this enzyme also do not exclude the diagnosis. If the level of ALT is elevated at least 1.5 times, that is the criterion for enrollment in a clinical trial, but ALT has only 51% sensitivity for the diagnosis of NASH [1,107]. In accordance with this information, there is also the fact that 11–30% of patients with a biopsy-confirmed NASH have a normal ALT value [107,108,109]. Previous studies have not confirmed an association between the level of elevation of transaminases and the diagnosis of NASH, the degree of fibrosis, or the degree of inflammation [110]. The significance of other serum biomarkers in distinguishing NASH from NAFLD was also examined. For example, cytokeratin 18, which is a marker of hepatocyte apoptosis is the only confirmed biomarker for NASH, but it is not in commercial use [13]. This parameter, as already mentioned, indicates hepatocyte apoptosis, which may be the end effect of lipotoxicity [111]. There are also clinical scores and indexes (Fibrosis-4 index, NAFLD fibrosis score, APRI) that allow physicians to estimate a degree of patient’s fibrosis without biopsy, but none of them is recognized for diagnosis of NASH [12,14,15,16]. The NAFLD score, which is designed for NAFLD, is valuable for the exclusion of progressive fibrosis with a 90% sensitivity [14,16]. The Fibrosis-4 index includes values of ALT, AST, platelet count, and patent age, and it is used for NAFLD and NASH [15,112].

Noninvasive evaluation of NAFLD and NASH includes ultrasound, CT scan, magnetic resonance imaging, and transient elastography [1,105]. Ultrasound does not represent the method of choice for diagnosing NAFLD and NASH. One study showed that ultrasound can overlook the diagnosis in as many as 22% of cases. In the case of coexistence of steatosis and fibrosis, which may be the case with NASH, the diagnosis can be even more difficult [113]. CT scan without use of contrast involves exposing the patient to radiation and costs more but does not provide any more precise data compared to ultrasound, so its use as a screening method is controversial [12]. In contrast, magnetic resonance imaging represents the most sensitive imaging method for assessment of hepatic steatosis, and it has 92% to 100% sensitivity and 92% to 97% specificity, but its limitation is cost [12]. Measuring elastic shear wave spreading is possible with transient elastography (FibroScan) or with magnetic resonance elastography. These two methods are accepted as the most sensitive for diagnosis and monitoring degree of fibrosis until and once in the state of cirrhosis [13,114]. However, the gold standard for diagnosis of NAFLD and NASH is liver biopsy, and it is the only recognized method for differentiating simple steatosis and NASH [2,115]. Given the risks and limitations it carries, a liver biopsy is not recommended for all patients with suspected NAFLD and NASH. Patients at high risk for development of NASH are ones with associated metabolic syndrome, elevated aminotransferases, older age, and Hispanic ethnicity [1]. In these individuals, it is justified to perform liver biopsy.

## 5. Association between Insulin Resistance, Type II Diabetes Mellitus, Cardiovascular Diseases, and NAFLD/NASH

Several studies have shown a link between insulin resistance (IR), type II diabetes mellitus (DMT2), and NAFLD/NASH [21,22,23]. Insulin is a hormone produced by the β cells of the Langerhans islets of the pancreas. It has a role in maintaining homeostasis of blood glucose levels. Insulin acts through a receptor that has tyrosine kinase activity and, after interacting with it, stimulates anabolic reactions in target cells [23]. Insulin resistance includes hyperinsulinemia, hyperglycemia, elevated free fatty acids (FFAs), and elevated pro-inflammatory cytokines. Skeletal muscles are often identified as the site of IR onset [21,116]. In skeletal muscle, IR reduces glucose uptake as well as its transformation into glycogen. In the liver, under normal conditions, the release of insulin after a meal inhibits the process of glycogenolysis and gluconeogenesis, which limits the leap of postprandial glycemia. However, in IR, this mechanism is disrupted [23]. In adipose tissue, IR causes lipolysis, which increases the influx of FFA by port circulation in the liver [23]. In addition, IR leads to adipose tissue dysfunction and the release of pro-inflammatory cytokines that play a role in the progression of NAFLD to NASH or the progression of pre-existing NASH. Therefore, IR has been identified as a major factor in the progression of NAFLD in NASH [117,118]. Hyperinsulinemia and hyperglycemia lead to the activation of hepatocyte stellate cells that play a role in hepatic fibrogenesis, and the feature of NAFLD progression in NASH is the occurrence of fibrosis [118]. Some meta-analyses have shown that NAFLD occurs in about 55.5% of patients with DMT2 and NASH in about 37.3% [119]. DMT2 contributes to the progression of NAFLD in NASH as confirmed by numerous studies [18,119]. The explanation lies in the fact that lipotoxicity has been proven to be present in the metabolic syndrome, which occurs with high frequency among patients with DMT2 and may represent a predisposing factor for developing NAFLD and NASH.

Disorders of lipid metabolism are common for NAFLD and cardiovascular diseases. Most patients with NASH, even those with uncomplicated NAFLD, have an increased cardiovascular risk [30,120]. The increase in visceral and ectopic adipose tissue increases the influx of FFA into the liver, which stimulates the accumulation of lipids but also de novo lipogenesis. This triggers a range of plasma lipoprotein disorders and leads to overproduction of very-low-density lipoproteins (VLDL), which is accompanied by decreased levels of high-density lipoprotein (HDL) and a predominance of low-density lipoprotein (LDL) particles [121,122]. In addition, certain lipoproteins activate Toll-like receptors, which form part of the innate immune system, and thus activate inflammatory pathways that cause vascular inflammation, endothelial damage, and increased risk of atherosclerosis and cardiovascular disease [123]. Because of that, NAFLD stimulates the development of vascular damage and atherosclerosis [121,124]. Patients with NAFLD are at higher risk of developing clinically manifested atherosclerosis, such as, most commonly, coronary heart disease [125]. NASH is an independent predictor of the occurrence of high-risk atherosclerotic plaques and therefore the occurrence of adverse coronary events [126]. Accumulation of ectopic adipose tissue can also affect the heart, predominantly the epicardium [127,128]. Epicardial ectopic adipose tissue produces a larger amount of pro-inflammatory cytokines, which damage the microvascular system of the heart and activate pro-fibrotic pathways. The consequence of these changes is the progression or development of coronary heart disease, arrhythmia, and heart failure. A large number of studies confirm the importance of NAFLD in increasing the risk of arrhythmia, primarily atrial fibrillation and ventricular arrhythmias [129,130,131]. This risk is particularly high in people with type II diabetes mellitus [130,132]. About 40–70% of patients with NAFLD have arterial hypertension [133].

Insulin resistance, which is often associated with NAFLD and NASH, can lead to disorders of the renin–angiotensin–aldosterone system, fibrinolysis disorders, and cardiac autoimmune neuropathy and thus lead to systolic and diastolic dysfunction, arrhythmias, and endothelial dysfunction [121]. A large number of patients with NAFLD develop signs of myocardial remodeling, especially left ventricular remodeling, and this condition is often accompanied by ventricular systolic or diastolic dysfunction [134]. Patients with NASH and advanced fibrosis and patients with NAFLD who also have DMT2, are at particular risk [103,129].

## 6. Treatment

**Figure 2:**
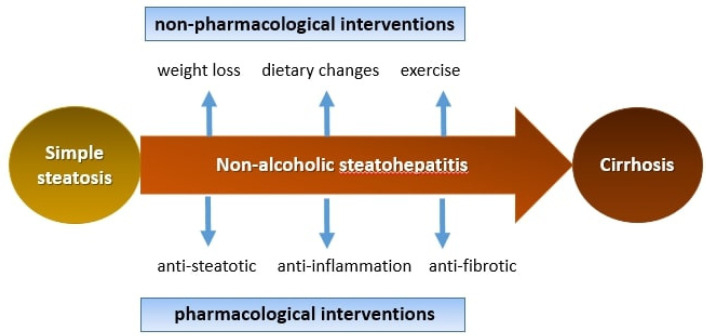
NASH treatment options.

Currently, there is no specific therapy for NAFLD and NASH, but there are several therapeutic options that are divided depending on whether they involve the use of pharmacological agents (Figure 2).

### 6.1. Lifestyle Modifications

The first choice in treatment are lifestyle modifications, i.e., non-pharmacological interventions. These include weight loss, dietary changes, and physical exercise [135]. Loss of at least 10% of total body weight is required for regression of fibrosis within NASH [136,137,138], 7–10% loss may be sufficient to reduce hepatic inflammation, and a loss of about 5% reduces the content of liver fat by about 30%. Weight loss alone cannot reduce the increased cardiovascular risk in these patients [139]. It is advised to patients with NAFLD and NASH to have diet low in fructose, saturated fat, simple carbohydrates, and sugars [140]. The Mediterranean diet, which is rich in monounsaturated fatty acids and/or eicosapentaenoic acid (EPA) and docosahexaenoic acid (DHA), which reduce cell membrane lipid oxidation and inflammation and enhance endothelial function, has been shown to be suitable [121,141]. In a study conducted by Ryan et al., the magnetic resonance imaging of the liver after six weeks of the Mediterranean diet showed significant improvement in hepatic steatosis [142]. The same effect was observed in a prospective randomized CENTRAL study [139]. In terms of physical activity, patients with NAFLD and NASH are recommended high-intensity exercise more than moderate-intensity exercise, as it has better effects in their case [143,144,145].

Since the above measures do not lead to satisfactory results in all patients, there was a need to expand the number of therapeutic ranges to pharmacological methods. Given the complexity of the pathophysiology of NAFLD and NASH, it is necessary that adequate therapy has anti-steatotic, anti-inflammation, and anti-fibrotic effects [79].

### 6.2. Drugs That Are in Use for Diabetes Mellitus Treatment

Metformin reduces hyperglycemia by affecting the process of gluconeogenesis in the liver and increases the sensitivity of peripheral tissues to insulin [33,146], but its role in the treatment of NAFLD has not been proven. However, the use of metformin in patients who have developed cirrhosis as a result of NASH and are also diabetics is associated with reduced mortality and less frequent occurrence of hepatocellular carcinoma [147,148].

Sodium-glucose cotransporter 2 inhibitor dapagliflozin in combination with omega-3 carboxylic acids significantly reduces the fat content in the liver and reduces the concentration of markers of hepatocyte damage [149]. Another drug from this group is empagliflozin, which is effective in reduction of liver steatosis in patients with DMT2 [150].

Peroxisome proliferator-activated receptors (PPARs), which are divided into three classes, regulate glucose and lipid homeostasis [151]. One of the classes, called thiazolidinediones, can reduce the degree of inflammation and fibrosis of the liver [4]. A representative of this class is pioglitazone, which is PPARG agonist. Its positive effects on NASH have been shown in several studies. For example, a meta-analysis conducted on patients with NASH who are diagnosed by a liver biopsy showed that pioglitazone can reduce fibrosis and cause NASH resolution even if patients who do not have DMT2 [152]. Moreover, pioglitazone may reduce cardiovascular risk in patients with NAFLD and DMT2 [153]. All these effects are related to modulation of lipid metabolism and insulin sensitivity as key roles of PPAR gamma. That is why there is the possibility of using pioglitazone in the treatment of DMT2 and insulin resistance, which are the conditions related to NAFLD [154].

Dipeptidyl peptidase-4 antagonists are used in the treatment of DMT2, and some studies have shown the possible benefit of their use in NAFLD patients, but further studies are needed to fully investigate this effect [155].

Glucagon-like peptide-1 (GLP-1) agonists inhibit glucose-dependent insulin secretion and secretion of glucagon. This way, they control homeostasis of plasma glucose. GLP-1 also activates GLP-1 receptor in hypothalamus, which results in weight loss (through satiety enhancement) [156]. Two representatives of this group are liraglutide, which has been shown to be effective in weight loss and reduction of liver steatosis, and semaglutide, which is effective in reducing steatosis in NASH, but its effect on already developed fibrosis has not been proven [157,158,159]

### 6.3. Antioxidant

The role of vitamin E in reducing NASH activity was also investigated, but opinions were divided due to insufficiently studied effect and possibly increased risk of developing malignancy during long-term substitution [2,5]. Vitamin C in dose of 800 mg/day was more effective than placebo in treating patients with NASH and without diabetes mellitus. It has shown histological improvement in this group of patients [79].

### 6.4. Probiotics and Symbiotics

Some studies have shown that patients with NAFLD and NASH may potentially have benefit from the use of probiotics and symbiotics [160].In children, the onset of NASH has multifactorial etiology. This includes genetic background and environmental factors (low physical activity, unhealthy diet, etc.). The idea of a possible hereditary occurrence of NASH arose due to the well-known fact that NASH occurs in several families in multiple members but also due to differences in the frequency of this condition in members of different ethnic and racial groups. Data from animal models suggest the importance of different genes, microRNAs, and proteins as well as the detection of de novo genes that are potential candidates for heritability [161]). Of particular importance are the genes responsible for regulating the intrahepatic concentration of FFA and oxidative stress in NAFLD [161]. On the other hand, it is shown that in children with NAFLD, a combination of several probiotic bacterial strains that are included in VSL#3 formulation showed notable advance in NAFLD and weight loss [162]. Moreover, a randomized clinical trial from Kobyliak et al. showed that multi-strain probiotics have beneficial effect on level of pro-inflammatory cytokines, aminotransferases, and liver fat content [160].

### 6.5. Other Innovative Therapeutic Possibilities

Some synthetic derivates of human bile acid chenodeoxycholic acid are effective in treatment of NASH although their primary purpose is to treat cholestatic liver disease. The representative of this group is obeticholic acid (OAC), which is a Farnesoid X receptor antagonist [119,163]. Obeticholic acid has been shown to be effective in reducing NASH without the deterioration of fibrosis [119]. Obeticholic acid has several adverse effects, of which pruritus and rise of low-density lipoprotein cholesterol level are the most common [164]. To prevent these side effects, Farnesoid X receptor antagonists that are not acid derivatives from bile acids were synthesized. For example, the most efficient of them is Tropifexor, which has positive effect on reduction of ALT and gamma-glutamyl transverase (GGT) levels and also reduction of liver steatosis [165].

The influence of other substances in the treatment of NAFLD and prevention of its progression in NASH was investigated: lipogenesis inhibitors, thyroid hormone beta agonists, fibroblast growth factor-19 analogue, and the others. However, the use of all these drugs is still under investigation, and so far, none of them has been approved for use in the treatment of NAFLD and NASH [79,158,166].

## 7. Conclusions

NASH is the inflammatory form of NAFLD, and besides that, it is characterized by the development of fibrosis. NASH is often associated with conditions characterized by disorders of lipid and glucose metabolism. Due to the increase in visceral and ectopic adipose tissue, there is an increased influx of FFA in the liver, and de novo lipogenesis is stimulated. Lipotoxicity is manifested by damage to hepatocyte organelles and release of pro-inflammatory cytokines, inflammation, and oxidative stress. These mechanisms are also mentioned in the pathogenesis of diseases that are associated with the development of NAFLD/NASH, such as IR, DMT2, and cardiovascular diseases. Patients with NASH and associated IR or DMT2 are at increased risk of adverse cardiovascular events. Arterial hypertension, arrhythmias, and structural heart damage are common in patients with NAFLD and NASH. To date, no specific therapy has been approved to treat NAFLD and prevent its progression to NASH. The only effective treatment at the moment is lifestyle modification, which includes weight loss, diet changes, and physical exercise. Based on the knowledge about the pathophysiology of NAFLD and NASH, numerous studies examine the use of innovative drugs that should reduce the risk of fibrosis progression and the occurrence of permanent complications. The important effects to be achieved in patients with NASH are anti-inflammatory and antioxidant. Pioglitazone, probiotics, and symbiotics have shown a role in reducing the degree of inflammation, while vitamin E and vitamin C are well-known antioxidants. Given the high incidence of NAFLD in the world’s population and the fact that progression in NASH increases the possibility of lasting consequences, such as liver cirrhosis, new studies are needed to investigate preventive and therapeutic options.

## Data Availability

Not applicable.

## Abbreviations

US	ultrasound
MR	magnetic resonance
DMT2	diabetes mellitus type 2
AF	atrial fibrillation
VT	ventricular tachycardia
